# Cold Forming of Al-TiB_2_ Composites Fabricated by SPS: A Computational Experimental Study

**DOI:** 10.3390/ma13163456

**Published:** 2020-08-05

**Authors:** Elad Priel, Nissim U. Navi, Brigit Mittelman, Nir Trabelsi, Moshe Levi, Sergey Kalabukhov, Shmuel Hayun

**Affiliations:** 1 Department of Mechanical Engineering, Center for Thermo-mechanics and Failure of Materials, Shamoon College of Engineering, Beer-Sheva 84100, Israel; brigit@post.bgu.ac.il (B.M.); nirtr@sce.ac.il (N.T.); 2 Department of Materials, Nuclear Research Center Negev (NRCN), Beer-Sheva 84190, Israel; 3 Department of Materials Engineering, Ben-Gurion University of the Negev, Beer-Sheva 8410501, Israel; moshe2@post.bgu.ac.il (M.L.); kalabukh@bgu.ac.il (S.K.)

**Keywords:** AMCs, ductile failure, finite elements, SPS

## Abstract

The mechanical response and failure of Al-TiB_2_ composites fabricated by Spark Plasma Sintering (SPS) were investigated. The effective flow stress at room temperature for different TiB_2_ particle volume fractions between 0% and 15% was determined using compression experiments on cylindrical specimens in conjunction with an iterative computational methodology. A different set of experiments on tapered specimens was used to validate the effective flow curves by comparing experimental force–displacement curves and deformation patterns to the ones obtained from the computations. Using a continuum damage mechanics approach, the experiments were also used to construct effective failure curves for each material composition. It was demonstrated that the fracture modes observed in the different experiments could be reproduced in the computations. The results show that increasing the TiB_2_ particle volume fraction to 10% results in an increase in material effective yield stress and a decrease in hardening. For a particle volume fraction of 15%, the effective yield stress decreases with no significant influence on the hardening slope. The ductility (workability) of the composite decreases with increasing particle volume fraction.

## 1. Introduction

Aluminum Matrix Composites (AMCs) are attractive structural materials [1] due to the unique characteristics of aluminum and aluminum alloys matrix combined with the characteristics of the ceramic particles. The Al matrix has a high strength-to-weight ratio compared with ferrous materials, as well as high ductility, good corrosion resistance, machinability, and processing flexibility, while the ceramic particles have exceptionally high strength. By changing the volume fraction of the ceramic particles in the composite along with their size and shape, it is theoretically possible to tailor the effective mechanical properties to a specific engineering application. This ability to control the effective mechanical properties makes AMCs attractive in many engineering applications where decreased ductility and increased strength are commonly required.

Commonly used methods for AMC synthesis are casting (stir, squeeze and more), powder metallurgy, pressure infiltration, spray atomization, and co-deposition [2,3]. In Spark Plasma Sintering (SPS), a mixture of particle and matrix materials, in powder form, is compressed in a closed die under constant high pressure and simultaneously sintered using an electric current that flows through the powder and tools [4].

In many cases, following the AMC synthesis, a subsequent forming process is applied, such as rolling, extrusion, or forging. This stage is required in order to achieve further consolidation [5,6], final shape [7], enhance particle distribution uniformity [8], or improved engineering properties [9].

As particles are usually added to the aluminum matrix in order to obtain increased strength, they are commonly made of ceramic materials, such as SiC, Al_2_O_3_ and B_4_C. TiB_2_ is also a promising candidate for aluminum reinforcement [10]. The volume fraction of the ceramic particles is expected to have a significant influence on the mechanical properties. For example, in [11] the stress–strain relation in tension of A380 + 5% TiB_2_ was shown to have a clear strengthening effect (in both yield and ultimate tensile stress) by the TiB_2_ particles, compared to the A380 alloy alone. In [12] it was shown that the increase of TiB_2_ content from 5% to 10% in sintered Al-TiB_2_ preforms decreased its strain to failure. The study in [13] compared A359 with 0%, 10%, 20% and 30% particle volume fractions of SiC. In quasi-static compression conditions, the A359 with 10% SiC material exhibited a significantly lower hardening compared to the reference matrix material. A higher SiC content does not affect the hardening significantly. Additionally, an increase in the yield strength is observed for higher SiC content. However, the relation between the particle volume fraction and the mechanical properties is not always direct. In an AMC system of AA7075 with SiC particles, it was shown that a small addition of SiC particles (1%) improved the material’s strength, whereas the addition of 5% caused the strength properties to deteriorate compared tothe original matrix material [14].

**Figure 1:**
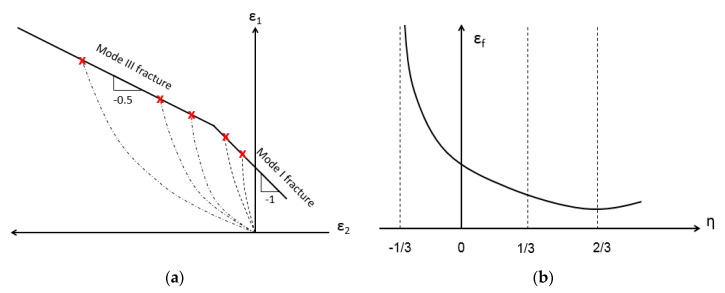
An example of a fracture initiation curve in the 2D principal strain space (**a**) and the effective strain–stress triaxiality space (**b**).

In metal forming processes, it is imperative to ensure that the process is complete without local damage to the material (such as cracking). In sheet metal forming, it is customary to use a 2D diagram of the workability in the principal strain space [15]. This contains a curve over which fracture initiates in the material. The same idea may be implemented in bulk metal forming for the case of surface cracks as was demonstrated in [16]. In [16] it was shown, based on bulk-forming tests, that this curve has a bi-linear form, with a straight segment of slope −0.5 associated with mode III fracture and a straight segment of slope −1 associated with mode I fracture. Figure 1a shows a schematic representation of such a curve in the 2D principal strain space. Each of the dashed curves in Figure 1a represent the collection of values of the ε1 to ε2 relation along with the entire experiment (progression with time) at the fracture initiation location. The curves end at the instant of fracture initiation (denoted in red). Similar data from different experiments (the changes may be geometrical or in experimental parameters, such as difference in lubrication) provides multiple dashed curves and their ending point. The fracture mode is also provided by the experimental data (the fracture mode is visually characterized). Each of the linear sections is constructed from the fracture initiation points (in red). It should be noted that this observation was made based on experiments on non-composite materials.

In recent years, a ductile fracture initiation criterion for bulk material forming based on the space of equivalent strain to fracture and stress triaxiality [17] has gained wide acceptance. Triaxiality, usually denoted by η, is defined as $\eta =\frac{\sigma_{P}}{\bar{\sigma }}$, where $\sigma_{P}$ is the hydrostatic stress and $\bar{\sigma }\;$ is the equivalent (Von-Mises) stress. A schematic example of a $\varepsilon_{f}\left(\eta \right)\;$ curve can be found in Figure 1b.

In [15] it was shown that using computational models and the 2D forming limit curve it is possible to construct the equivalent strain to fracture vs. stress triaxiality curve (i.e., to obtain a $\varepsilon_{f}\left(\eta \right)$ curve from the $\varepsilon_{1}\left(\varepsilon_{2}\right)$ curve).

The goal of the current study is to investigate the influence of particle volume fraction on the effective flow stress and failure modes of an Al-TiB_2_ composite produced using SPS.

Following this introduction, the manuscript contains four additional sections. Section 2.1 and Section 2.2 describe the experimental and computational methodology for both determining the effective flow stress and constructing the material failure curves. Section 2.3 is devoted to the computational model and the iterative process for extracting the effective flow curves and for modeling ductile failure. Section 3 presents validation of the computational models and the results of experimental computational process. Section 4 discusses the results, including the influence of particle volume fraction. A summary and the conclusions of the study are provided in Section 5.

## 2. Materials and Methods

**Figure 2:**
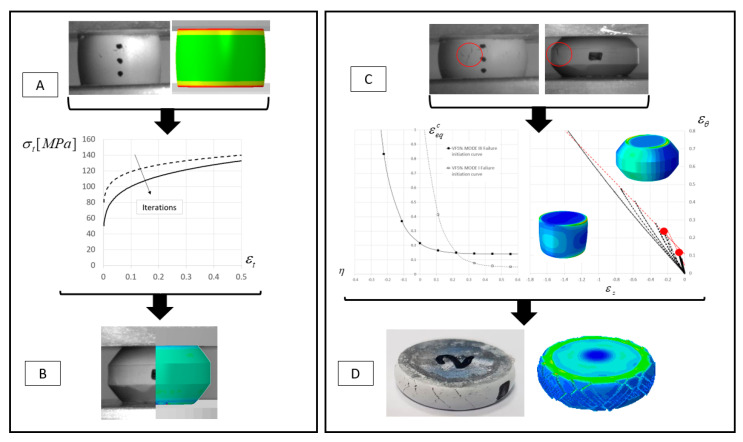
Schematic representation of the research methodology used in this study: Determination of flow stress for each particle volume fraction (**A**), validation of flow stress curves (**B**), construction of failure initiation curves (**C**), validation of failure initiation curves (**D**).

The goal of the current study is to investigate the influence of TiB_2_ volume fraction on the effective mechanical response and failure modes of an Al-TiB_2_ composite. The effective flow stress is determined using compression tests on cylindrical specimens in conjunction with finite element analysis. Failure modes are investigated using a continuum damage approach for which the effective failure initiation curves are constructed based on the experimental methodology outlined in [16]. An outline of the research methodology is presented in Figure 2.

### 2.1. Material and Specimen Preparation

Al (>99.9%, Glentham, GX6811, Wiltshire, UK) and TiB_2_ (Grade F, H.C. Stark GmbH, Giessen, Germany) powders were used to obtain 0, 5, 10 and 15 vol.% of Al-TiB_2_ powder mixes. The particle size distribution of the original powders was analyzed using the QICPIC dynamic image analysis system (Sympatec@GmbH, Clausthal-Zellerfeld, Germany). Details about the measurement procedure is described in [18] and results are given in Table 1.

Proper weight proportions from the original powders were mixed (total mix volume of ~210 cm^3^) using a POWTEQ M10 tumbler (at 25 rpm) in a 1 L container (100 mm diameter). For the 10 and 15 vol.% TiB2, 10 alumina balls (a diameter of 21 mm each, ~205 gr total weight) were added to improve powder mix homogeneity. X-ray diffraction (XRD) (Rigaku RINT 2100 Tokyo, Japan with Cu Kα radiation with λ = 1.54 Å), with operating parameters of 40 kV and 30 mA in the 2θ range from 20° to 80°, with a step size of 0.02° and a scan step time of 1 s, was used to determine the mixing powder homogeneity (samples were taken from the upper, middle and bottom portions of the powders mix) and phase content. The XRD patterns (see Figure 5) of the powders were analyzed to confirm Al-TiB_2_ composition using a whole pattern fitting approach (MDI Jade 2019 version 7.7 software, Livermore, CA, USA). Samples from the sintered billets were mechanically ground and polished down to 1 μm for microstructural characterization. Scanning electron microscope (SEM, JEOL JSM-7400F, Tokyo, Japan) combined with energy-dispersive X-ray spectroscopy (EDS, Thermo Fisher Scientific, Waltham, MA, USA) detector was used for morphology characterization and to determine sample’s local composition and homogeneity. Back-scattered electron (BSE) imaging and the open-source software Paint Net (v4.2.1, dotPDN LLC, San Francisco, Ca, USA) were used to determine the distribution of the principal phases.

Typical powder mixing conditions and the composition that was obtained from the XRD analysis are shown in Table 2.

**Figure 3:**
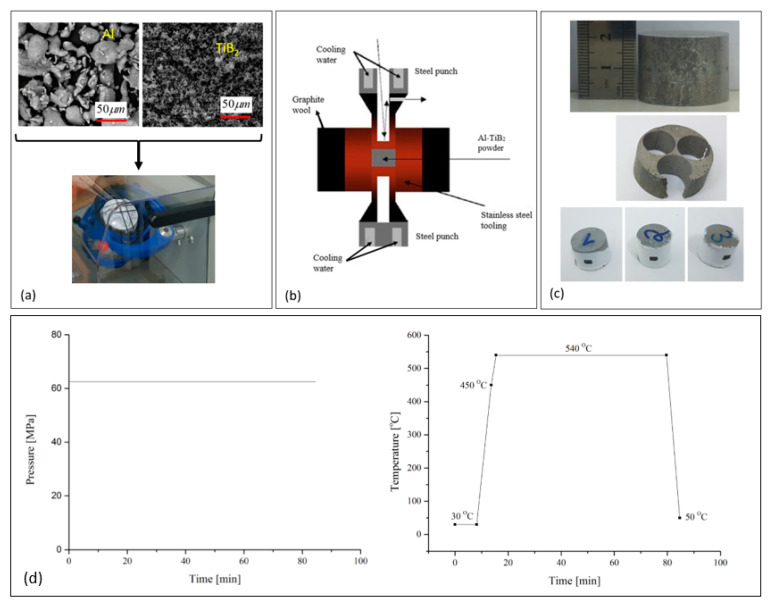
Specimen fabrication using the SPS method. (**a**) The powder mixture used for the specimens. (**b**) A schematic description of the SPS method. (**c**) An example of a specimen obtained from the SPS. (**d**) The SPS process pressure and temperature.

The powder was then transferred to a Spark Plasma Sintering (SPS) (FCT HP D10, FCT System GmbH, Frankenblick, Germany) [19,20] system to obtain a sintering of the billet with an outer diameter of 30 mm and a height of 20 mm, at 540 °C and constant pressure of 62.5 MPa. Heating of the powder mix was conducted in two stages, first with a heating rate of 76 °C/min between 30 and 450 °C, and then with a rate of 50 °C/min from 450 to 540 °C. Cooling from 540 °C to 50 °C was performed with a cooling rate of 98 °C/min. Specimens for compression experiments were cut from the sintered billets using electric discharge machining (EDM). Sample preparation and sintering procedure are illustrated in Figure 3.

**Figure 4:**
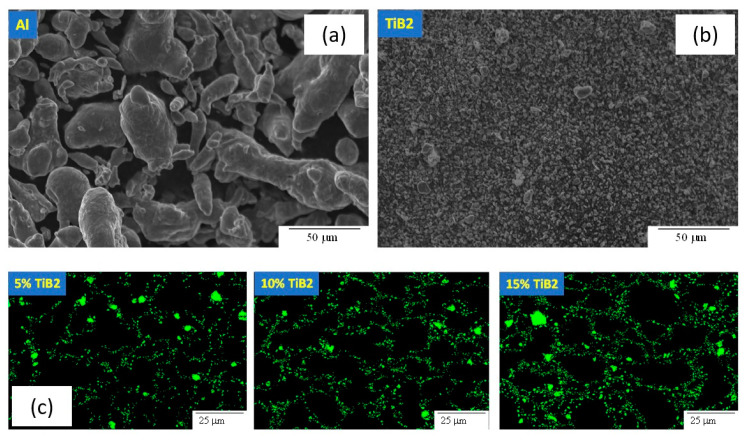
SEM images of Al powder (**a**) and TiB_2_ powder (**b**), and BSE images of 5, 10 and 15 vol.% of Al-TiB_2_ composites fabricated by SPS (Green color denotes TiB_2_ particles) (**c**).

Representative SEM micrographs of the original powders and the resulting composite microstructure can be seen in Figure 4. It is evident from Figure 4 that, due to the large size difference between the Al particles and the TiB_2_ particles, the microstructure is characterized by large particles of Al with numerous small TiB_2_ particles arranged around the Al particle boundaries.

**Figure 5:**
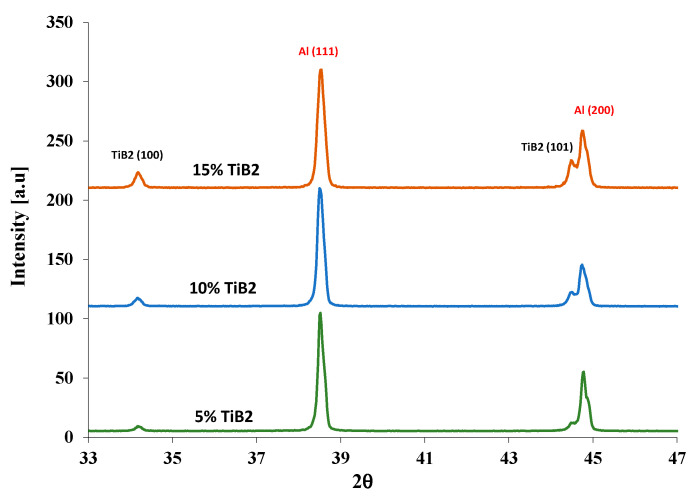
Typical normalized XRD diffractograms of the Al-TiB_2_ powders.

Typical XRD scans of the Al-TiB_2_ powders, within a range of 2θ = 33–47° are shown in Figure 5, indicating an increase in the TiB_2_ peak intensities with the increase of TiB_2_ volume fraction.

The mixed powders do not contain any significant impurities (not shown here).

### 2.2. Experimental Setup

The experiments were divided into two sets. In the first set, compression experiments were conducted on cylindrical specimens with different TiB_2_ particle volume fractions and used to determine the effective flow stress for the different compositions. The flow stress was obtained by an iterative experimental/computational procedure similar to the one described by the authors in [21,22].

**Figure 6:**
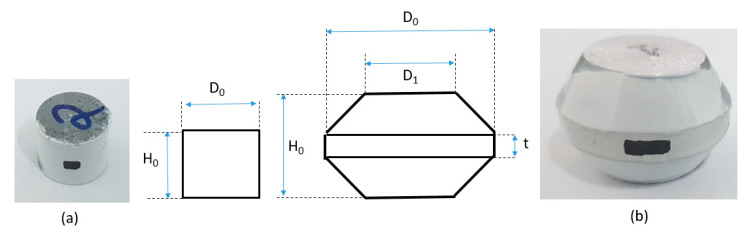
Experimental specimens used in the current study: cylindrical (**a**), tapered (**b**).

Another set of compression experiments was performed using tapered specimens (see Figure 6). This group of experiments was used to both validate the effective flow stress determined from the first stage of experiments and to construct, with the results of the first set of experiments, the effective failure initiation curves for each composition.

The specimen geometries for each experimental set are presented in Figure 6.

The dimensions of the different specimen geometries used in the study are provided in Table 3.

**Figure 7:**
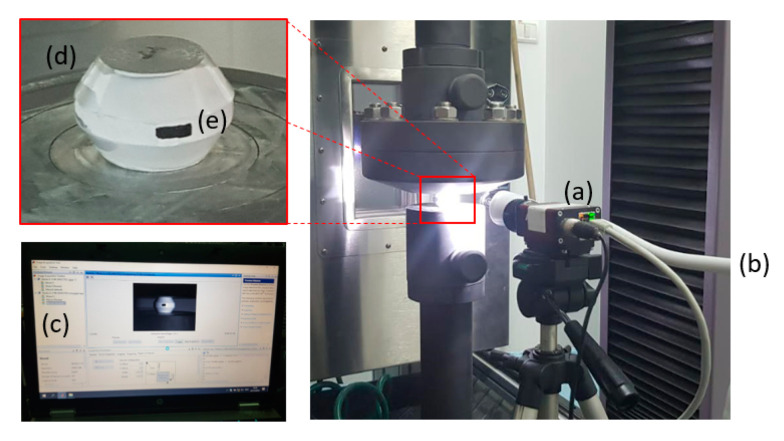
Experimental setup: high-resolution camera (**a**), lighting source (**b**), MATLAB code for image processing (**c**), test specimen (**d**), markings for tracking specimen deformation (**e**).

The mechanical tests were conducted on a 300 KN electro-mechanical machine. The specimens were loaded at a constant ram velocity of 1 mm/min until failure was attained. The experiments were also monitored by a high-resolution camera in order to capture the specimen deformation history for model validation and also to identify the time and location of the initial specimen failure. An example of the setup can be seen in Figure 7.

Experiments on both types of specimen geometries showed the expected specimen barreling due to friction between the specimen and tools (as no lubrication was used during the experiments). These friction forces result in a stress field which is no longer uniaxial. As a consequence, numerical methods that take these shear stresses into account are required in order to determine the effective composite flow curves from the experiments as discussed by the authors in [21,22]. The flow–stress results will be presented in Section 3.

### 2.3. The Computational Models

The commercial finite element code ABAQUS/Standard 6.14 [23] was used to construct 3D models of the compression tests taking system symmetry into account. The effective plastic response of the AMC composite (for all particle volume fractions) was assumed to follow linear elastic isotropic Hook’s law in the elastic range and the J_2_ (Von-Mises) yielding criterion with isotropic strain hardening in the plastic range.

The forming tools were assumed to be much stiffer than the specimens, and therefore were modeled as rigid bodies. Contact between specimen and tools was enforced using the penalty method with a coulomb-type friction model. The friction coefficient was assumed to be similar to the one between cast Al and tool steel, which was determined in a past study by the authors [21]. Verification of the computational models was conducted by standard mesh convergence tests in which the mesh was refined until convergence in both local values (such as plastic strain) and global values (such as strain energy) was obtained (see Appendix A).

**Figure 8:**
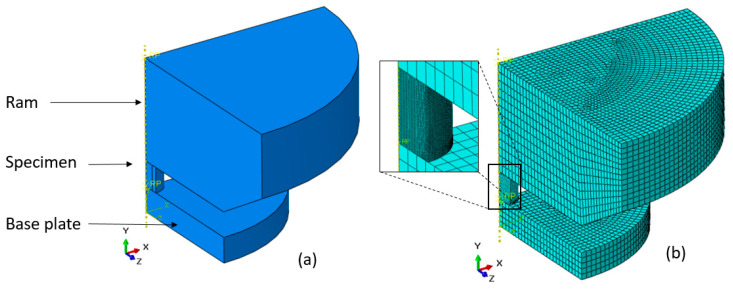
Example of model geometry (**a**) and mesh (**b**) for the cylindrical specimens.

The final computational mesh used for analysis of the experiments was constructed of 114,000 hexahedral elements for the specimen and 22,000 elements for the tools. Displacement boundary conditions which match the experimental ram displacement were prescribed to the ram while the base plate was clamped. An example of the model geometry and mesh for the cylindrical specimens can be seen in Figure 8.

#### Continuum Damage Model

To model ductile failure and fracture, several quantities have to be determined: the point of damage initiation in the material, and the damage evolution (until the material reaches a state in which the point can no longer sustain any load). The damage initiation is assumed to be governed by a critical value of the effective plastic strain ${\bar{\varepsilon }}_{D}\left(\eta \right)$, which depends on the stress triaxiality η. A state variable $\omega_{D}$ is computed at each integration point during the deformation process with $\omega_{D}=1$, indicating that damage has been initiated.

(1) $$\omega_{D}=\int^{\;}\frac{d{\bar{\varepsilon }}_{D}}{{\bar{\varepsilon }}_{D}\left(\eta \right)}$$

Once damage has been initiated, it is postulated, as in [24], that a critical energy density $G_{c}$ is required to completely damage a material point. The energy density G can be computed as:

(2) $$G=\int_{0}^{{\bar{\varepsilon }}_{f}}\bar{\sigma }\left(\bar{\varepsilon }\right)d\bar{\varepsilon }$$

with $\bar{\varepsilon },\bar{\sigma }$ being the equivalent strain and stress, and ${\bar{\varepsilon }}_{f}$ denoting the strain at failure.

From Equation (2) a scalar damage variable *D* can be defined as:

(3) $$D=\frac{1}{G_{c}}\int_{0}^{{\bar{\varepsilon }}_{f}}\bar{\sigma }\left(\bar{\varepsilon }\right)d\bar{\varepsilon }$$

with *D* = 0 indicating no damage and *D* = 1 indicating full failure.

From a computational perspective, local damage approaches are mesh dependent due to strain localization. To alleviate mesh dependency, a local characteristic length is introduced in Equation (3), similar to the concepts proposed in [25].

(4) $$D=\frac{1}{G_{f}}\int_{{\bar{\varepsilon }}_{D}}^{{\bar{\varepsilon }}_{f}}L\bar{\sigma }\left(\bar{\varepsilon }\right)d\bar{\varepsilon }\;\;\;\;\;$$

with $G_{f}\;$ being the energy density per unit area and L being the element size. Equation (4) can also be formulated as:

(5) $$D=\frac{1}{G_{f}}\int_{0}^{{\bar{u}}_{f}}L\bar{\sigma }\left(\bar{u}\right)d\bar{u}$$

**Figure 9:**
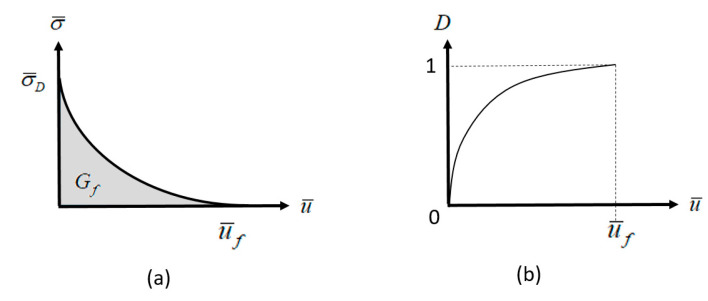
Methods for modeling damage evolution: (**a**) stress–equivalent plastic displacement relation; (**b**) damage parameter as a function of equivalent plastic displacement.

Here, $\bar{u}$ is defined as the equivalent plastic displacement and ${\bar{u}}_{f}$ is the critical plastic displacement at full failure. It is important to note that the value of $\bar{u}$ is only relative to the state at damage initiation. To model the damage evolution, one of two possible definitions may be used: either the stress vs. displacement relation following damage initiation needs to be provided, or the scalar damage variable *D* must be defined, as a function of the equivalent plastic displacement (see Figure 9).

## 3. Results

### 3.1. Effective Flow Stress Curves as a Function of Particle Volume Fraction

Using the methodology outlined in [21], the flow curves at room temperature for each particle volume fraction were determined and fit to the Holloman relationship of the form:

(6) $$\sigma =K\varepsilon^{n}$$

The Holloman model constants for each case are summarized in Table 4.

**Figure 10:**
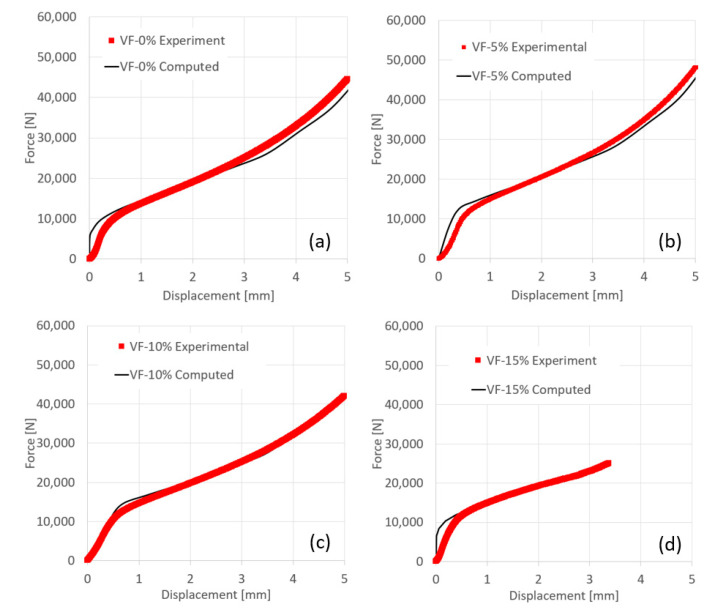
Comparison between computed (black line) and experimental (red dots) force displacement curve. (**a**) Al-0% TiB_2_; (**b**) Al-5% TiB_2_; (**c**) Al-10% TiB_2_; (**d**) Al-15% TiB_2_.

In Figure 10, a comparison between the computed and experimental force displacement curves using the effective flow stress curves determined in this study is presented.

**Figure 11:**
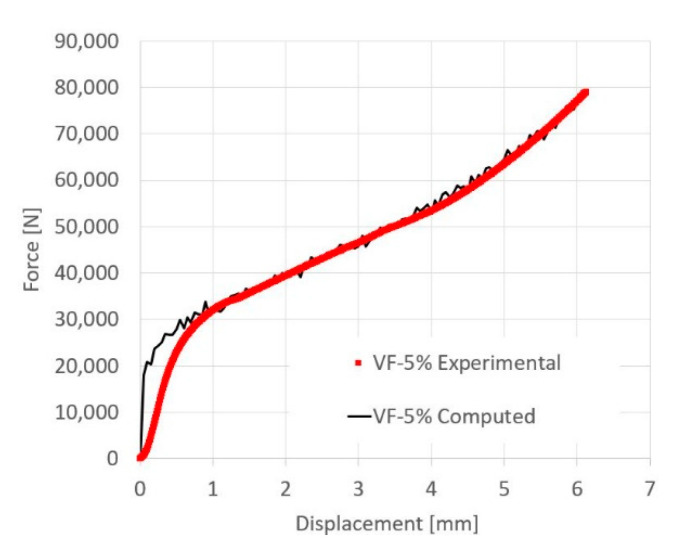
Comparison between computed (black line) and experimental (red dots) force displacement curves for a tapered specimen containing 5% TiB_2_ particle volume fraction.

**Figure 12:**
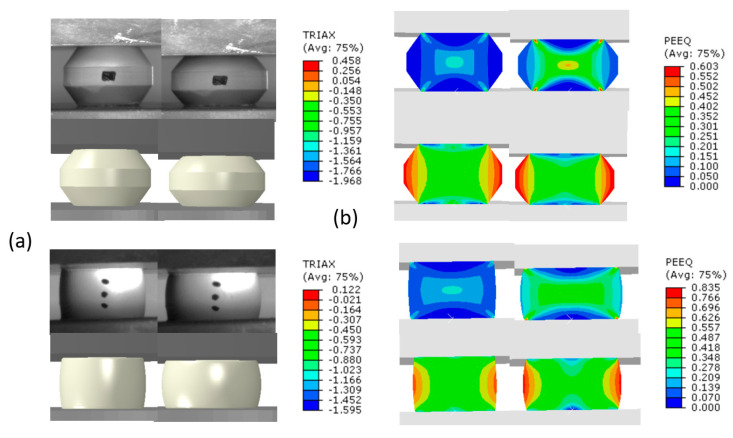
Computed vs. experimental deformation for the tapered and cylindrical specimen containing 5% TiB_2_ volume fraction prior to specimen failure (**a**); a cut across the specimen showing distribution of equivalent plastic strain (PEEQ) and triaxiality (TRIAX) (**b**).

To validate the effective flow stress curves, the material model parameters determined from the first set of experiments were used to model the second set of experiments conducted on the tapered specimens. As an example, a comparison between the computed and measured force displacement curve for a tapered specimen containing 5% TiB_2_ volume fraction is shown in Figure 11. A comparison of computed and observed deformation, triaxiality and equivalent plastic strain for the same specimen is shown in Figure 12, at two different stages of the experiment.

It can be observed from Figure 12a that the predicted and observed contour of the deformed specimens for the same ram displacement is similar. It is also shown that just prior to the initial failure (Figure 12b), high plastic strain values and high negative triaxiality values can be observed at the specimen center, while the edges of the specimen display low plastic strain values with high positive triaxiality values. It is also evident that the positive triaxiality values which develop on the specimen surface are higher in the tapered specimen.

### 3.2. Determination of Damage Initiation Curves and Influence of Damage Evolution Curves

**Figure 13:**
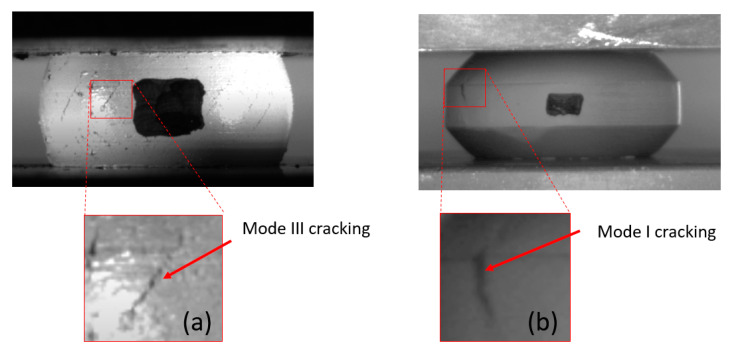
Fracture modes for the different specimen types: Mode III for the cylindrical specimens (**a**); and Mode I for the Tapered specimens (**b**).

As previously mentioned, the fracture envelope $\varepsilon_{1}\left(\varepsilon_{2}\right)$ in the 2D strain domain may be used in conjunction with FE models to obtain $\varepsilon_{f}\left(\eta \right)$. It was shown in [15] that the $\varepsilon_{1}\left(\varepsilon_{2}\right)$ curve has a bi-linear form, and that the straight segment associated with mode III fracture has a slope of −0.5, while the straight segment associated with mode I fracture has a slope of −1. This work was based on experimental data of several metals (although not on composite materials). The two geometries used in the current experiments (cylindrical, C and tapered, T) result in two points on the $\varepsilon_{1}\left(\varepsilon_{2}\right)$ graph, each associated with a different failure mode, so it is possible to construct the bi-linear failure curve in the 2D strain domain. For this loading case, the principal strains on the specimen surface are $\varepsilon_{\theta }$ and $\varepsilon_{Z}$. In Figure 13, the different fracture modes observed for each specimen type are shown.

**Figure 14:**
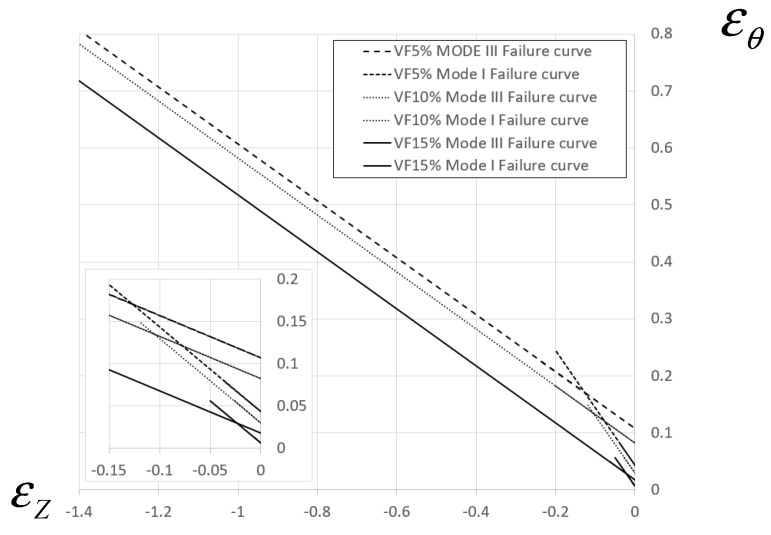
Failure curves in the principal strain space for Mode III and Model I fracture.

It should be noted that following failure initiation, some mode I cracks in the tapered specimens branched into Mode III cracks. The moment and point of failure initiation were determined from experiments (the tests were filmed at a high acquisition rate of 30 frames per second), and the values of principal strains $\varepsilon_{\theta }$ and $\varepsilon_{Z}$ were obtained from the computational models. The resulting bi-linear failure curve for every material composition is presented in Figure 14.

**Figure 15:**
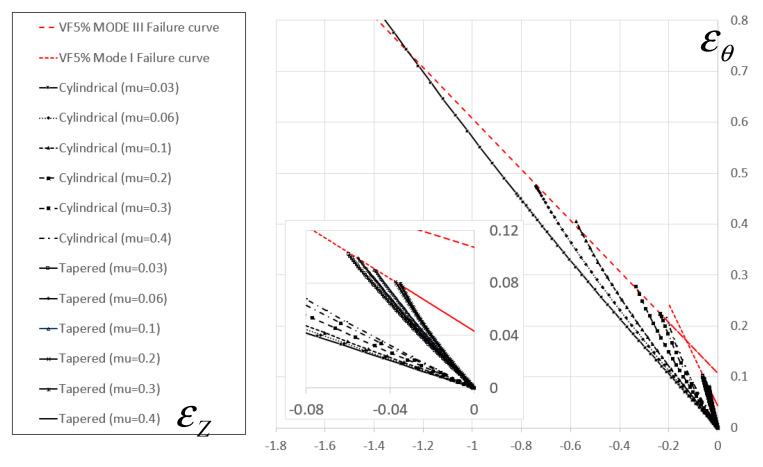
Principal strain path for different loading cases for specimens containing 5% volume fraction of TiB_2_ particles.

Once the fracture envelope in the 2D strain domain was determined, fracture initiation under different values of triaxiality were obtained numerically. This was achieved by computing the strain and stress fields for compression experiments using different values of friction coefficients at the interface between the tools and the specimen, within the range μ=0.03−0.4. An example of the principal strain path for the different cases is shown in Figure 15 for the 5% volume fraction composition.

**Figure 16:**
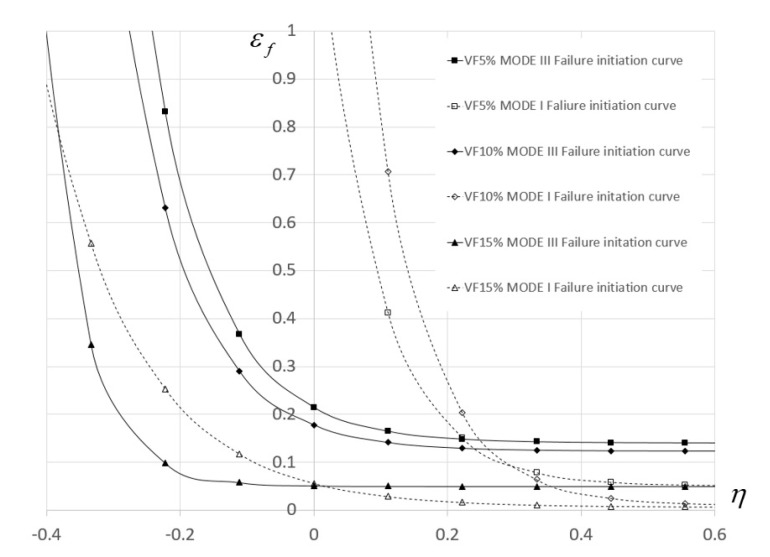
Damage initiation curves for different TiB_2_ particle volume fractions.

For each such case the equivalent plastic strain and triaxiality history at the location of failure can be computed up to failure initiation. Assuming that the relation $\varepsilon_{f}\left(\eta \right)$ takes the form of $\varepsilon_{f}\left(\eta \right)=D_{1}+D_{2}\exp\left(D_{3}\eta \right)$ for each mode of fracture, the set $D_{1},D_{2},D_{3}$ was determined for each material composition by minimizing the target function $F\left(\varepsilon_{eq},\eta ,\varepsilon_{f}\right)=1-\omega_{d}\left(\varepsilon_{eq},\eta ,\varepsilon_{f}\right)$ using non-linear regression. The relation $\varepsilon_{f}\left(\eta \right)$ for the different TiB_2_ particle volume fraction is presented in Figure 16.

The values of $D_{1},D_{2},D_{3}$ for each material composition and fracture mode are provided in Table 5.

It is important to note that the curves in Figure 16 do not directly define the value of $\varepsilon_{eq}$ at failure for a specific value of η. The curves $\varepsilon_{f}\left(\eta \right)$ are only used to define damage initiation by computing the damage initiation parameter $\;\omega_{d}$. Only for specific loading cases in which the value of η is constant up until failure initiation (like the case of uniaxial tension) does the value of $\varepsilon_{eq}$ equal $\varepsilon_{f\;}$ at failure initiation.

**Figure 17:**
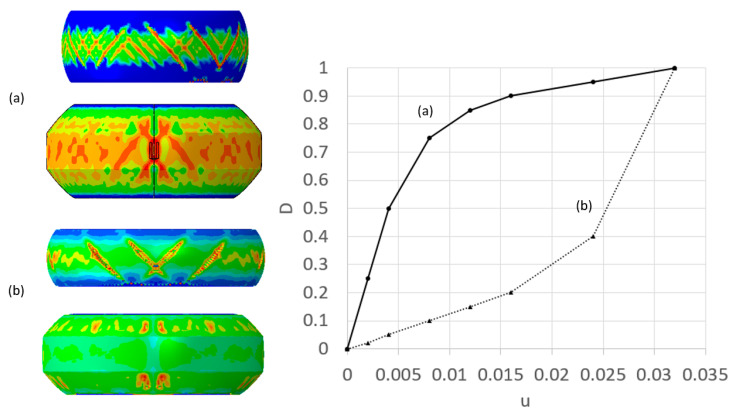
Computed failure mode obtained for different damage evolution curves: fast propagation of damage (**a**) and slow propagation of damage (**b**); color indicates the damage parameter D.

The damage initiation curves determined in the previous section are not sufficient for modeling specimen failure propagation, as this is governed by damage evolution. The experiments conducted on the cylindrical and tapered specimens were used to determine the damage evolution curve for each composition. This was conducted using an iterative approach similar to the one used to determine the flow–stress curves. In this case, by changing the function $D\left(\bar{u}\right)$, the resulting computed fracture patterns were compared to the experimentally observed fracture patterns. Figure 17 shows that a different path of damage evolution curve $D\left(\bar{u}\right)$ results in a different fracture patterns even if one uses the same damage initiation curve.

As demonstrated in Figure 17, a damage evolution curve in which local damage accumulates slowly as a function of local displacement (curve (b) in Figure 17) results in a decrease in the number of cracks which develop across the specimen surface. For fast accumulating damage curves (curve (a) in Figure 17), the damage paths can intersect forming crisscross patterns across the specimen surface.

The discrete values of the evolution curves are provided in Table 6.

**Figure 18:**
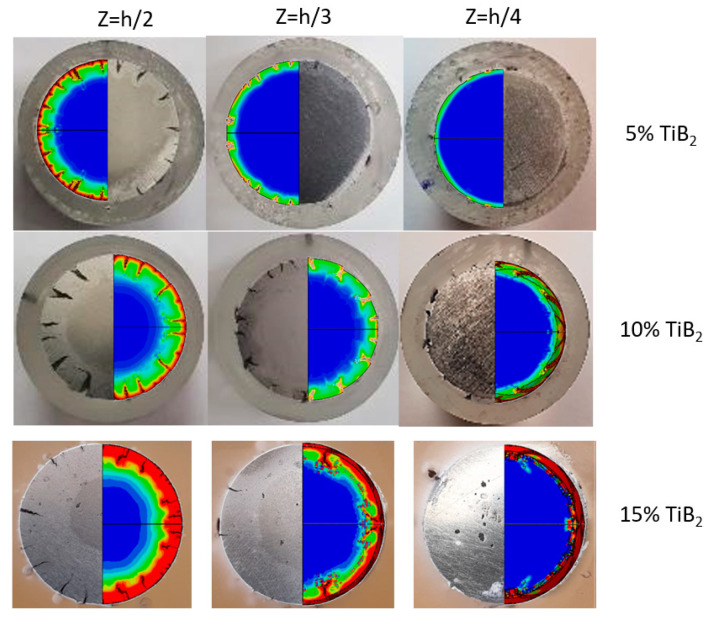
Computed and observed crack formation at different heights along the cylindrical specimen axis, Colors indicate value of the damage initiation parameter $\omega_{d}$ (see Equation (1)).

Using the above $D\left(\bar{u}\right)$, the fracture mode observed in the experiment was reproduced in the FE computations for both the cylindrical and tapered specimens. As an example, in Figure 18, the observed cracks at different heights along the cylindrical specimen axis are compared to the computed crack formation.

**Figure 19:**
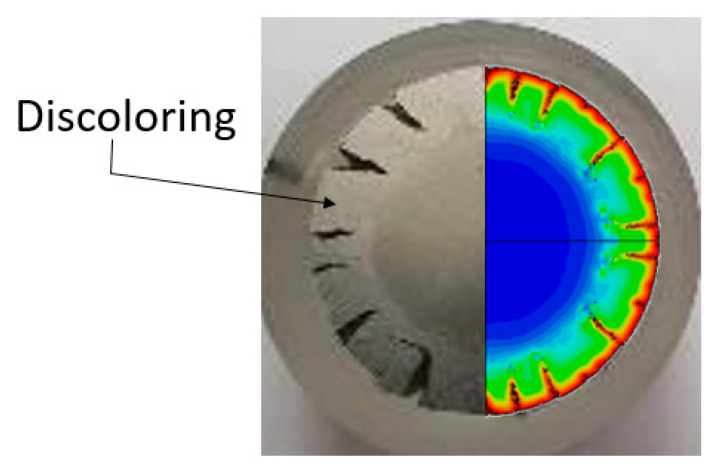
Some discoloring contours observed in the metallurgical examination which can be correlated with the computed damage initiation parameter.

As demonstrated in Figure 18, the damage model is able to reproduce cracking mode observed in the experiments for all material compositions. It is interesting to note that as a result of cutting, polishing and etching of the specimen surface, the outer segment appears to be of a slightly different brightness. This area may be correlated with the damage distribution patterns, as shown in Figure 19.

Future work will inspect these areas for evidence of micro-cracking or microvoids, which could be directly related to the initiation of ductile damage.

## 4. Discussion

The current study investigated the influence TiB_2_ particle volume fraction has on two aspects: the effective flow stress of the Al-TiB_2_ composite; and failure initiation and evolution at room temperature. In the case of the Al-TiB_2_ composites produced by SPS, the TiB_2_ particles are not chemically bonded to the Al particles. This fact greatly influences both the effective flow stress and failure initiation as explained below.

### 4.1. Effective Flow Stress of Al-TiB_2_

It has been demonstrated that even though no explicit consideration of the microstructure is incorporated in the description of the constitutive response, the effective flow stress determined from the iterative computational experimental method is sufficient to accurately capture deformation modes and forces for different specimen geometries. Nevertheless, the accuracy of an effective description of the flow stress may diminish if smaller specimens (such as thin foils) or very large ceramic particles are considered, so that the particle size can no longer be considered very small with respect to the specimen size.

The effective flow stress of the Al-TiB_2_ composite can be described by two parameters: (a) the effective yield stress, (b) the effective hardening slope. The results demonstrate (see Table 4) that addition of 5% and 10% volume fraction of TiB_2_ particles increases the effective yield stress of the material. Following this, at some point, further increase in particle volume fraction does not result in an increased yield stress but a slight decrease as shown when the volume fraction increased to 15%. With respect to the strain hardening slope, the addition of the ceramic particles decreases the strain hardening parameter n. The addition of up to 10% TiB_2_ particles decreases the hardening slope significantly; however, further increase of particle volume fraction does not have a significant influence on the hardening slope.

As the micrographs show (see Figure 4), each Al particle is surrounded by TiB_2_ particles. It is assumed that the initial increase in yield stress can be attributed to the fact that the ceramic particle obstructs initial dislocation movement between adjacent Al particles. When the volume fraction of TiB_2_ particles is further increased, plastic deformation is no longer limited to dislocation movement in the Al grains but is enabled by relative sliding of Al particles because of the TiB_2_ relative movement across the interface. This mechanism may be the reason for the decrease in yield stress, which is observed at large TiB_2_ particle volume fractions. This mechanism also explains the apparent decrease in effective strain hardening coefficient. With increasing TiB_2_ volume fraction, more of the Al particle surface area is surrounded by TiB_2_ particles. This results in more instances of relative sliding between Al particles during deformation which leads to a decrease in observed effective strain hardening.

### 4.2. Failure and Fracture of Al-TiB_2_

The current study uses continuum damage mechanics concepts in order to investigate and model failure initiation and fracture modes of different compositions of Al-TiB_2_. The experiments show that failure mainly occurs by mode I (opening mode) and mode III (out of plane shear), as reported in past studies for Al-2024 and steel specimens [16]. The results demonstrate that increasing the volume fraction of the TiB_2_ particles decreases the effective ductility of the material. The decreased ductility influences the workability of the material at room temperature conditions (the ability to undergo cold forming). This is clearly evident from the effective failure curve in the principal strain space (Figure 14), as the area under the 2D failure curve can be directly linked to the material workability and it decreases monotonically for increasing particle volume fraction.

Ductile failure in metals is attributed to growth and coalescence of voids. In the case of the Al-TiB_2_ composites produced by SPS, the TiB_2_ particles are not chemically bonded to the Al particles. This implies that each TiB_2_ particle (or aggregate of particles) is essentially a void in the aluminum matrix. Increasing the particle volume fraction theoretically increases the number of voids within the matrix, thereby decreasing the material’s ductility. Increasing the number of particles also means that the Al particles are more loosely joined to one another.

These two phenomena are not incorporated directly in the continuum damage model used in this study; however, their theoretical influence on specimen failure can be represented using the continuum approach. First, an increase in particle volume fraction results in a decrease in the equivalent plastic strain required for damage initiation (see Figure 15) for both the low and high triaxiality range. Second, the fracture energy density per unit area (Gc) decreases for increasing TiB_2_ particle volume fraction (as evident from the function $D\left(\bar{u}\right)$). The reduction in fracture energy can be linked to the decrease in Al-Al particle interfaces.

It should be noted that it is not clear how any changes in TiB_2_ particle size or shape will influence the effective flow stress and effective failure curves obtained in this study. It is assumed that variations in particle size and shape will have some influence on the effective flow stress but that the main effect will be on the effective failure curves and effective fracture energy density. Such phenomena can be explored using a micro-macro computational approach, which will be considered in future work.

## 5. Conclusions

The mechanical response and failure of Al-TiB_2_ composites fabricated using SPS were investigated. The effective flow stress for different TiB_2_ particle volume fractions was determined using an iterative computational and experimental methodology. A different set of experiments (changing specimen geometry) was used to validate the effective flow curves by comparing experimental force displacement curves and deformation patterns to the ones obtained from the computations.

The computational models and experiments were also used to construct effective failure curves for each material composition. The experimental results were also used to calibrate the effective fracture energy density for final failure of a material point in the continuum damage framework. It was demonstrated that using a continuum damage mechanics approach, the fracture modes observed in the different experiments could be reproduced by the computational models.

The results show that increasing the TiB_2_ particle volume fraction up to 10% results in an increase in material effective yield stress and a decrease in hardening. For a particle volume fraction of 15%, the effective yield stress decreases with no significant influence on the hardening slope. The ductility (workability) of the composite decreases for increasing particle volume fraction. This result is evident from the decreasing value of strain to failure for the entire range of triaxiality values

## Figures and Tables

**Table 1:** Al and TiB_2_ particle distribution.

Cumulative Volume Distribution	TiB_2_ (µm)	Al (µm)
10 (%)	6	7
50 (%)	7	32
90 (%)	13	70

**Table 2:** Typical powder mixing conditions and characterization.

Nominal TiB_2_ vol.%	Mixing Period (h)	Additional Alumina Balls	Vol.% of TiB_2_ from XRD
0	-	-	-
5	48	-	5.3 ± 0.2
10	72	Yes	9.9 ± 0.7
15	96	Yes	14.0 ± 0.4

**Table 3:** Dimensions of the experimental specimens used in the study (notations as in Figure 6).

Specimen Type	Notation	H_0_ (mm)	D_0_ (mm)	D_1_ (mm)	t (mm)
Cylindrical	C	10	12.7	-	-
Tapered	T	17	25	17	5

**Table 4:** Effective flow stress model parameters as a function of particle volume fraction.

Particle Volume Fraction (%)	K (MPa)	n (-)	$\sigma_{y}\;(\mathrm{MPa})$
0	140	0.2	42
5	150	0.175	50
10	145	0.15	57
15	131	0.148	51

**Table 5:** Parameters of the damage initiation curve for each material composition and fracture mode.

Model Parameters	Mode I Fracture			Mode III Fracture		
	$D_{1}$	$D_{2}$	$D_{3}$	$D_{1}$	$D_{2}$	$D_{3}$
Al-5%TiB_2_	0.140	0.075	−10.0	0.05	1.3	−11.5
Al-10%TiB_2_	0.123	0.055	−10.0	0.01	2.5	−11.5
Al-15%TiB_2_	0.049	0.0013	−16.3	0.006	0.05	−7.2

**Table 6:** Values of damage evolution curves for each TiB_2_ particle volume fraction.

	Damage Parameter D	0	0.25	0.5	0.75	0.85	0.9	0.95	1
u_p_ (mm)	Al-5%TiB_2_	0	0.002	0.004	0.008	0.012	0.016	0.024	0.032
	Al-10%TiB_2_	0	0.001	0.002	0.004	0.006	0.008	0.012	0.016
	Al-15%TiB_2_	0	0.0001	0.0002	0.0004	0.0006	0.0008	0.0012	0.0016

**Figure A1:**
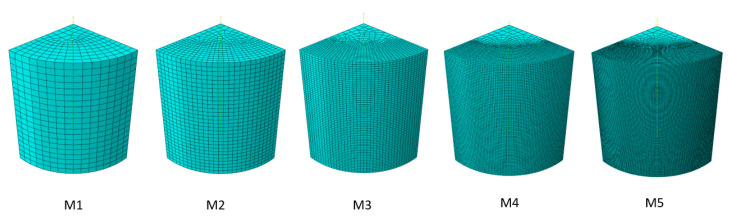
Mesh densities named M1-M5 used for solution convergence studies.

**Figure A2:**
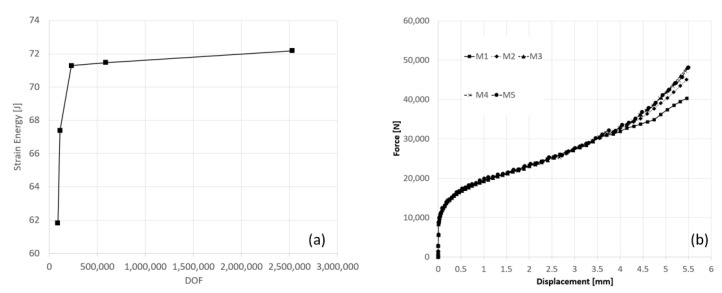
Convergence in global solution parameters: strain energy (**a**), force–displacement curve (**b**).

**Figure A3:**
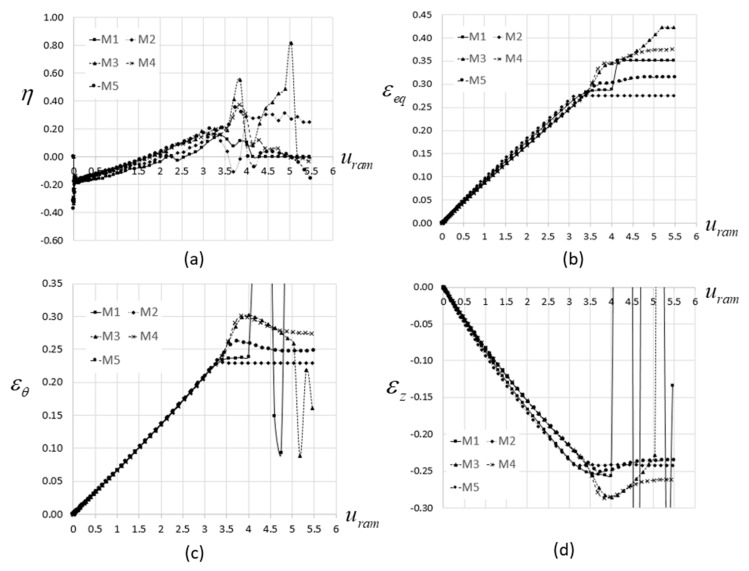
Convergence in local solution parameters extracted at the point on the specimen surface where initial failure was obtained: stress triaxiality (**a**), equivalent plastic strain (**b**), circumferential strain (**c**), axial strain (**d**).

**Figure A4:**
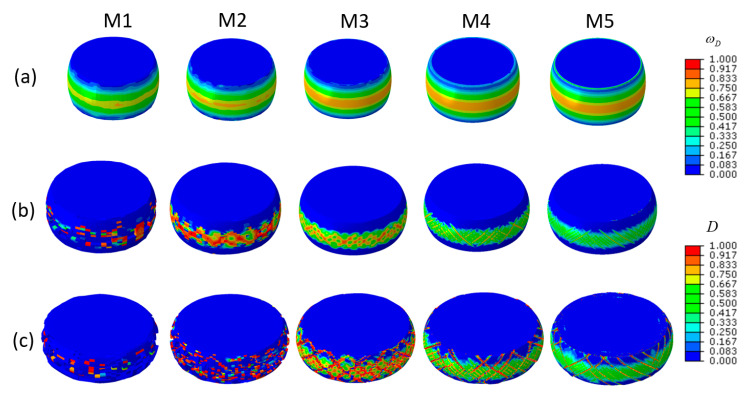
Convergence in damage initiation and damage evolution: damage initiation parameter prior to local failure initiation (**a**), damage evolution parameter D after initial failure (**b**), damage evolution parameter following cracking of the specimen surface (**c**).

## Appendix A

Verification of the computational models was conducted using slandered mesh convergence studies with both global and local field values examined. Emphasis was placed on insuring that the instant of damage initiation and damage evolution was not mesh dependent. The mesh for the forming tools remained constant, and only the mesh of the specimen was changed. It was verified that the tool mesh was sufficiently fine to resolve contact between the tools and specimen. For the specimen, five mesh densities were used, as shown in Figure A1 for the cylindrical specimen.

The number of degrees of freedom (DOF) ranged from 88425 (M1) to 2528753 (M5).

In Figure A2, convergence in global parameters such as maximum strain energy and force displacement curve is shown.

It can be seen that convergence in both strain energy and force–displacement curve is reasonable (less than 5% relative error) for mesh densities M3–M5.

For the local field variables, convergence in computed principal strains, stress triaxiality and equivalent plastic strain are shown in Figure A3.

Figure A3 demonstrates that up to local failure, which takes place at a ram displacement of about 3.5 mm in this example, convergence in local field variables is obtained for mesh densities M3–M5.

To examine the sensitivity of the continuum damage approach used for the mesh density, the ductile damage parameter $\;\omega_{D}$ and the damage evolution parameter *D* were plotted at different times during the failure of the specimen, as shown in Figure A4.

As shown in Figure A4, convergence is obtained in the computed instance of damage initiation for M3–M5 (distribution of damage initiation variable is similar). The mode III fracture mode is replicated by the computational model for mesh densities M3–M5. It can be seen that even though there is a great difference in mesh density between mesh M3 and M5, there is no change in the mode III crack patterns that appear on the specimen surface.
